# Dietary CoQ10 Ameliorates Age‐Related Megakaryocyte Dysfunction via COPS3‐Mediated Activation of Autophagy

**DOI:** 10.1111/acel.70726

**Published:** 2026-09-29

**Authors:** Yixuan Xu, Yu‐heng Mao, Fenglin Song, Fang He, Yueying Wu, CaiXia Wang, Minghan Wang, Shuangfeng Xie, Heyu Ni, Yan Yang

**Affiliations:** ^1^ School of Public Health (Shenzhen), Shenzhen Campus of Sun Yat‐Sen University, Sun Yat‐Sen University Shenzhen China; ^2^ Guangdong Engineering Technology Research Center of Nutrition Transformation, Sun Yat‐Sen University Shenzhen China; ^3^ Guangdong Provincial Key Laboratory of Food, Nutrition and Health, Sun Yat‐Sen University Guangzhou China; ^4^ School of Exercise and Health, Guangzhou Sport University Guangzhou China; ^5^ School of Food Science, Guangdong Pharmaceutical University Guangzhou China; ^6^ Department of Hematology Sun Yat‐Sen Memorial Hospital, Sun Yat‐Sen University Guangzhou China; ^7^ Department of Laboratory Medicine and Pathobiology University of Toronto Toronto Ontario Canada; ^8^ Department of Laboratory Medicine LKSKI‐Keenan Research Centre for Biomedical Science, St. Michael's Hospital, and Toronto Platelet Immunobiology Group Toronto Ontario Canada; ^9^ CCOA Therapeutics Inc Toronto Ontario Canada; ^10^ Department of Physiology University of Toronto Toronto Ontario Canada; ^11^ Canadian Blood Services Centre for Innovation Toronto Ontario Canada; ^12^ Department of Medicine University of Toronto Toronto Ontario Canada

**Keywords:** aging, autophagy, COPS3, CoQ10, megakaryocyte

## Abstract

Age‐related megakaryocyte (MK) dysfunction disrupts platelet production and may increase thrombotic risk, but whether coenzyme Q10 (CoQ10) restores MK homeostasis through autophagy remains unclear. Male C57BL/6J mice aged 1, 9, 15, 19, 21, or 23 months received CoQ10 for 12 weeks. Bone marrow MK proliferation, CD41/CD61 expression, DNA polyploidy, autophagy‐associated proteins, and platelet phenotypes were evaluated. H_2_O_2_‐induced senescent MEG‐01 cells were used for pharmacological modulation with rapamycin and LY294002, bafilomycin A1‐based autophagic flux assays, and COPS3 knockdown. CoQ10 enhanced MK proliferation, maturation, and polyploidization in aged mice. Changes in the LC3‐II/LC3‐I ratio and p62 in bone marrow MKs were consistent with enhanced autophagy. Proteomic analysis identified increased COPS3 abundance in MKs from CoQ10‐treated 24‐month‐old mice. In senescent MEG‐01 cells, responses to rapamycin and LY294002 supported the involvement of autophagy‐related signaling in the effects of CoQ10. CoQ10 increased COPS3 expression even under autophagy‐inhibitory conditions and produced greater bafilomycin A1‐sensitive LC3‐II accumulation, supporting increased autophagic flux. COPS3 knockdown attenuated CoQ10‐induced changes in autophagy‐associated markers and partially diminished its effects on MK maturation and polyploidization, indicating that COPS3 contributes to, but may not solely mediate, these responses. CoQ10 did not alter platelet counts in naturally aged mice but was associated with reduced platelet aggregation and activation. Together, these findings suggest that CoQ10 ameliorates age‐related MK dysfunction and platelet hyperreactivity, in part through COPS3‐associated autophagy regulation. These findings provide a mechanistic basis for further investigation of CoQ10 as a nutritional strategy for age‐related platelet dysfunction.

## Graphical abstract

Dietary CoQ10 improves megakaryocyte maturation and polyploidization and reduces platelet hyperreactivity in naturally aged mice, while experiments in senescent Meg‐01 cells demonstrate enhanced autophagic flux and identify COPS3 as a contributing, but not, mediator.

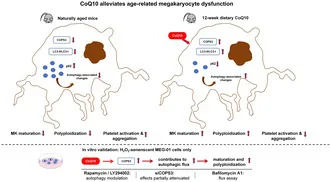

## 1 Introduction

The global trend of population aging has intensified the focus on age‐related hematological disorders, which represent a significant and growing health challenge (Falchetta et al. 2024; Guo et al. 2022; Jaul and Barron 2017). Hematopoietic aging is characterized by a functional decline across the blood system, with platelets exhibiting particular hyperreactivity that contributes to an elevated risk of cardiovascular diseases in the elderly (Rodgers et al. 2019). As the direct precursors of platelets, megakaryocytes (MKs) reside at the crux of this problem, playing a pivotal role in maintaining platelet homeostasis (Vainchenker and Raslova 2020; Sun and Shan 2019; Mansell et al. 2023). Aging impairs MK biology through both cell‐autonomous mechanisms and microenvironmental remodeling, processes that drive dysregulated apoptosis, compromise key quality‐control pathways, and ultimately lead to aberrant platelet production and function (Sun and Shan 2019; Schwertz and Middleton 2023; Yang et al. 2023). Despite the clear link between MK health and age‐related platelet abnormalities, the molecular drivers of MK dysfunction during natural aging remain incompletely defined.

Autophagy is a conserved intracellular degradation process essential for cellular quality control and metabolic homeostasis. Numerous studies have confirmed that reduced autophagic activity is closely associated with the progression of multiple age‐related diseases (Vainchenker and Raslova 2020; Palmer et al. 2025; Guo et al. 2022). Autophagy involves the conversion of soluble cytoplasmic LC3‐I into membrane‐bound LC3‐II to form autophagosomes, which subsequently interact with the autophagy receptor p62 and other cargo adaptor proteins (Ohsumi 2013; Runwal et al. 2019; Jung et al. 2019). The regulation of autophagy is intricately linked to ubiquitin signaling. The COP9 signalosome (CSN), an evolutionarily conserved multi‐subunit complex, regulates the activity of Cullin‐RING E3 ubiquitin ligases (CRLs) and has emerged as a critical modulator of diverse cellular processes, including autophagy. Notably, its core subunit, COPS3, has been shown to promote autophagic flux in cancer models and autophagic clearance in non‐cancer tissues, such as in cardiomyocytes (Niu et al. 2023; Zhang et al. 2018). In MKs, autophagy supports differentiation, polyploidization, and organelle development by clearing damaged components and providing energy. However, the role of autophagy in natural aging, particularly in the hematopoietic system and MK biology, remains completely unknown.

Coenzyme Q10 (CoQ10) is an endogenously synthesized, lipid‐soluble benzoquinone that is predominantly localized to the inner mitochondrial membrane. It transfers electrons from complexes I and II to complex III, thereby supporting oxidative phosphorylation and ATP production (Sun et al. 2019; Hargreaves 2021). Its reduced form, ubiquinol, also acts as an antioxidant that protects membrane lipids and mitochondrial components from oxidative damage. CoQ10 homeostasis and mitochondrial function may become impaired with aging, although these changes vary among tissues and species. CoQ10 supplementation has therefore been investigated in older populations and in several age‐related conditions. Human studies have reported potential improvements in oxidative stress, inflammation, fatigue, physical function, and selected cardiovascular outcomes. However, the overall evidence remains heterogeneous because of differences in study populations, CoQ10 formulations, doses, intervention durations, and outcome measures (Battino et al. 1990; Gutierrez‐Mariscal et al. 2021; Lenaz et al. 2007; Zhang et al. 2015; Xue et al. 2019). Although CoQ10 supplementation has been evaluated in age‐related conditions, its effects on bone marrow megakaryocytes during physiological aging remain largely unknown. Recent experimental studies suggest that CoQ10 may regulate autophagy, but whether this involves the COP9 signalosome has not been investigated.

In this study, we therefore hypothesized that dietary CoQ10 supplementation ameliorates age‐related MK dysfunction by restoring autophagic activity, and we further postulated that the COP9 signalosome, particularly COPS3, serves as a key mechanistic link. Using a naturally aged mouse model combined with unbiased proteomic screening and functional validation, we identified COPS3 as the critical mediator. We report for the first time that CoQ10 upregulates COPS3 expression, which in turn promotes autophagy, enhances MK maturation and polyploidization, and reduces apoptosis. Our findings not only elucidate a novel pathway of CoQ10 action but also establish COPS3 as a central regulator of proteostatic resilience in aging megakaryocytes, offering new insights into potential therapeutic strategies for mitigating platelet dysfunction in the elderly.

## 2 Results

### 2.1 Dietary CoQ10 Alleviates Oxidative Stress and Chronic Inflammation in Naturally Aged Mice

Klotho expression level is closely associated with aging and age‐related diseases, making it a key regulatory factor and a promising therapeutic target in anti‐aging research. Mice aged 1–9–15–19–21–and 23 months were administered a 12‐week dietary CoQ10 intervention until they reached 4–12–18–22–24–and 26 months of age, at which time experimental data were collected. In our experimental results, the Klotho protein expression level progressively declined with age in both the CoQ10 intervention group and the control group. However, its expression was modestly higher in CoQ10‐treated mice, reaching statistical significance at 24 months (*p* < 0.05) (Figure 1A). Oxidative stress assessment revealed an overall age‐related increase in oxidative stress. CoQ10 treatment reduced the expression of ROS in mice and increased the expression of T‐AOC (Figure 1B,C). Meanwhile, CoQ10 treatment reduced MDA levels and increased the expression or activity of antioxidant enzymes, including SOD, CAT, and GSH, in mice (Figure S1). Similarly, CoQ10 treatment suppressed systemic inflammation. Beginning at 12 months of age, CoQ10‐treated mice exhibited reduced plasma concentrations of IL‐6, TNF‐ɑ, and IL‐1β compared with controls (Figure 1D–F). At 22 months, IL‐6 and TNF‐ɑ levels were significantly lower with control (*p* < 0.05). IL‐1β levels were significantly decreased as early as 12 months (*p* < 0.05).

**FIGURE 1:**
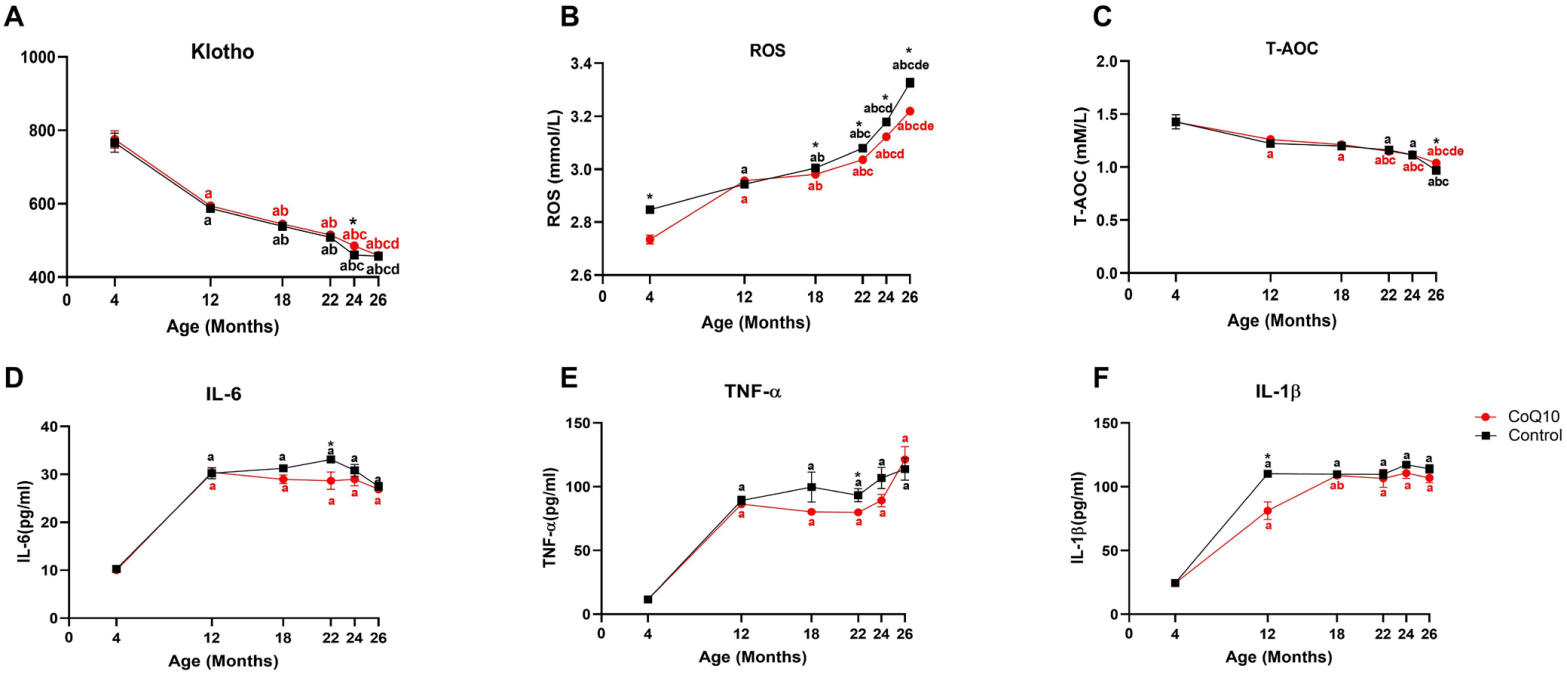
Coenzyme Q10 enhances circulating klotho levels and attenuates oxidative stress and inflammation in naturally aged mice. (A) Plasma klotho; (B) ROS; (C) T‐AOC; (D) IL‐6 levels in mouse plasma; (E) TNF‐ɑ levels in mouse plasma; (F) IL‐1β levels in mouse plasma (*n* = 7). Data are presented as mean ± SEM. a: *p* < 0.05 compared with 4‐month‐old group (4 M); b: *p* < 0.05 compared with 12‐month‐old group (12 M); c: *p* < 0.05 compared with 18‐month‐old group (18 M); d: *p* < 0.05 compared with 22‐month‐old group (22 M); e: *p* < 0.05 compared with 24‐month‐old group (24 M); *: *p* < 0.05 compared with the same age group.

### 2.2 Dietary CoQ10 Improves Proliferation, Maturation, and Differentiation of Bone Marrow Megakaryocytes in Naturally Aged Mice

After 12 weeks of CoQ10 supplementation, bone marrow MKs proliferation was evaluated using colony formation assays. MK colonies in the CoQ10‐treated group were larger and more numerous than those in controls, with significantly increased colony counts observed at 12, 16, and 18 months of age (*p* < 0.05) (Figure 2A,B). Flow cytometric analysis revealed that CoQ10 promoted MKs maturation and differentiation, as indicated by higher proportions of CD41^+^, CD61^+^, and CD41^+^CD61^+^ MKs relative to controls. Specifically, the proportion of CD41^+^ MKs increased significantly at 4 and 12 months, CD61^+^ MKs at 22, 24, and 26 months, and double‐positive CD41^+^CD61^+^ MKs at 22 and 24 months (*p* < 0.05) (Figure 3A–C). Ploidy analysis demonstrated no significant differences in diploid and tetraploid MK populations between groups. However, higher proportions of 16 N, 32 N, and 64 N MKs were observed in the CoQ10‐treated group, whereas 8 N cells predominated in controls (*p* < 0.05) (Figure 3D,E).

**FIGURE 2:**
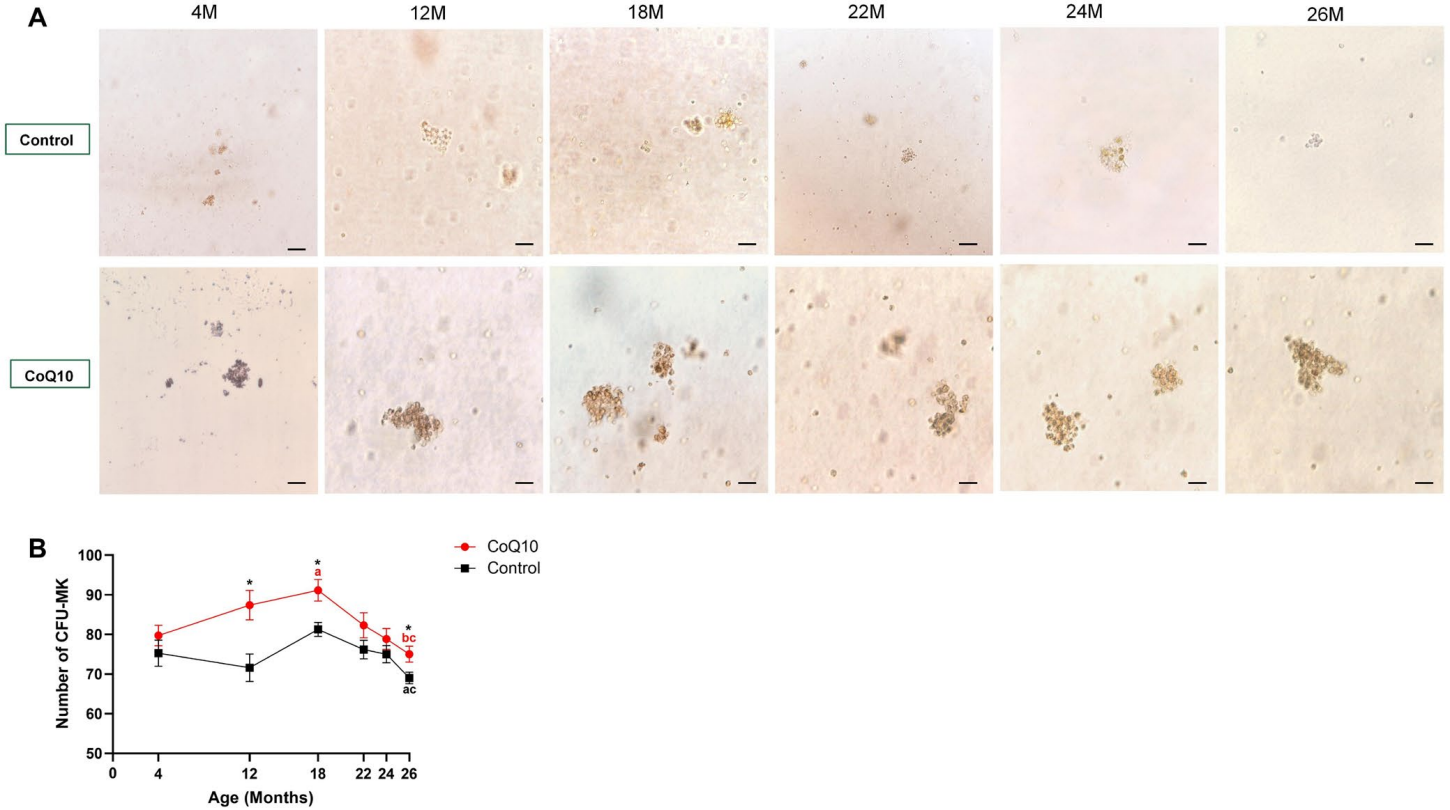
CoQ10 promotes proliferation and maturation in naturally aged mice. (A) CFU‐Meg colonies (magnification 20×; 50 μm); (B) The numbers of CFU‐Meg colonies (*n* = 6). Data are presented as mean ± SEM. a: *p* < 0.05 compared with 4‐month‐old group (4 M); b: *p* < 0.05 compared with 12‐month‐old group (12 M); c: *p* < 0.05 compared with 18‐month‐old group (18 M); *: *p* < 0.05 compared with the same age group.

**FIGURE 3:**
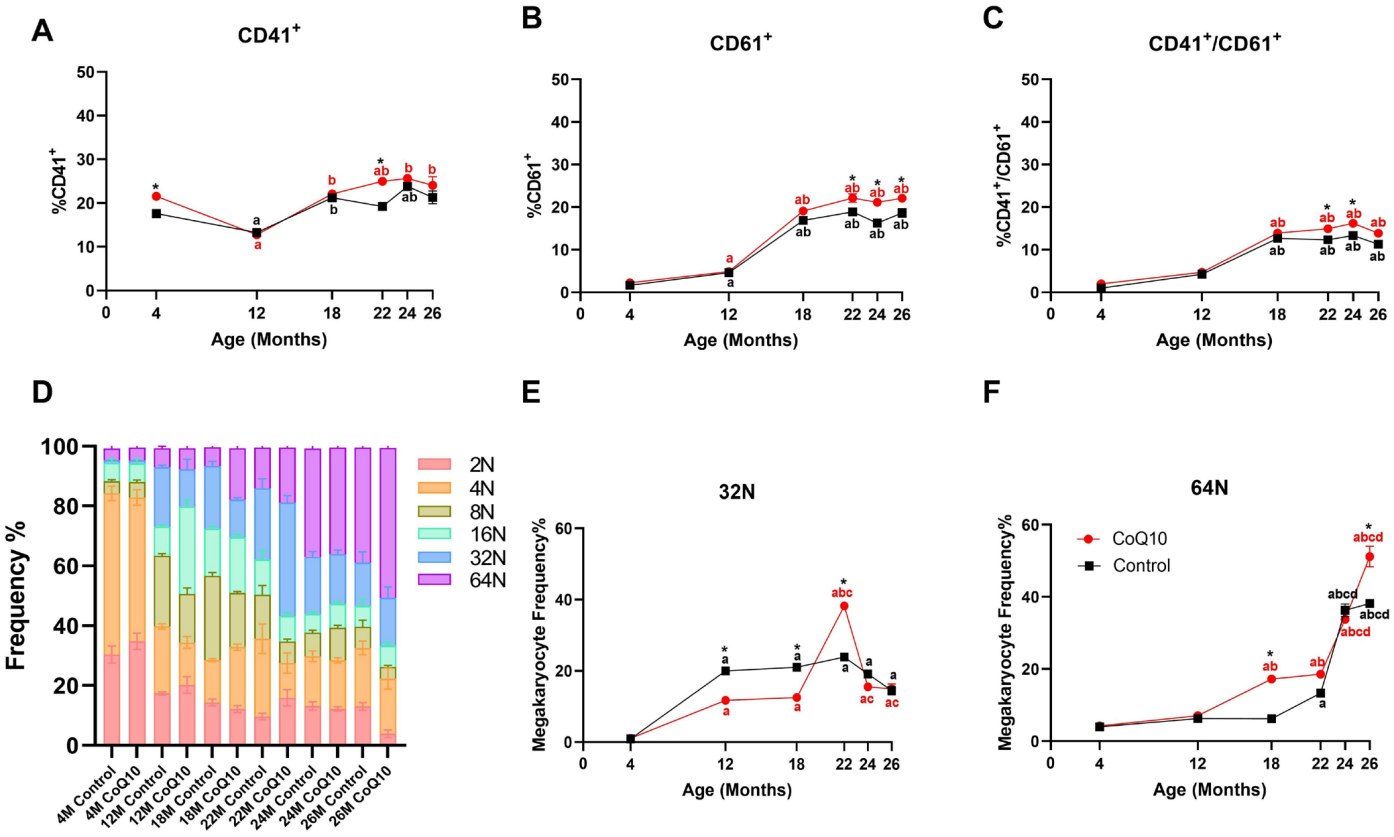
CoQ10 promotes maturation in naturally aged mice. (A–C) Flow cytometry was employed to detect and quantify the expression of CD41^+^, CD61^+^, and CD41^+^CD61^+^ in the bone marrow of naturally aged mice; (D) The DNA ploidy distribution (the percentage of cells with 2n–64n) was analyzed by flow cytometry (*n* = 6); (E–F) The DNA ploidy distribution (the percentage of cells with 32n and 64n) was analyzed by flow cytometry (*n* = 6). Data are presented as mean ± SEM. a: *p* < 0.05 compared with 4‐month‐old group (4 M); b: *p* < 0.05 compared with 12‐month‐old group (12 M); c: *p* < 0.05 compared with 18‐month‐old group (18 M); d: *p* < 0.05 compared with 22‐month‐old group (22 M); *: *p* < 0.05 Compared with the same age group.

### 2.3 Dietary CoQ10 Supplementation Upregulates COPS3 Expression and Enhances Autophagy in Megakaryocytes of Naturally Aged Mice

During natural aging, autophagy progressively declines, contributing to functional deterioration in multiple physiological systems. Analysis of autophagy‐related proteins in bone marrow megakaryocytes from naturally aged mice revealed that LC3II expression and the LC3II/LC3I ratio gradually decreased with age. In contrast, the CoQ10‐treated group exhibited significantly higher LC3II levels and LC3II/LC3I ratios at 12, 18, 22, 24, and 26 months compared with age‐matched controls (*p* < 0.05) (Figure 4A–D). Conversely p62 expression increased with age, whereas CoQ10 administration effectively suppressed its expression. Moreover, apoptosis assays demonstrated that CoQ10 markedly reduced megakaryocyte apoptosis at 22, 24, and 26 months (*p* < 0.05) (Figure 4E).

**FIGURE 4:**
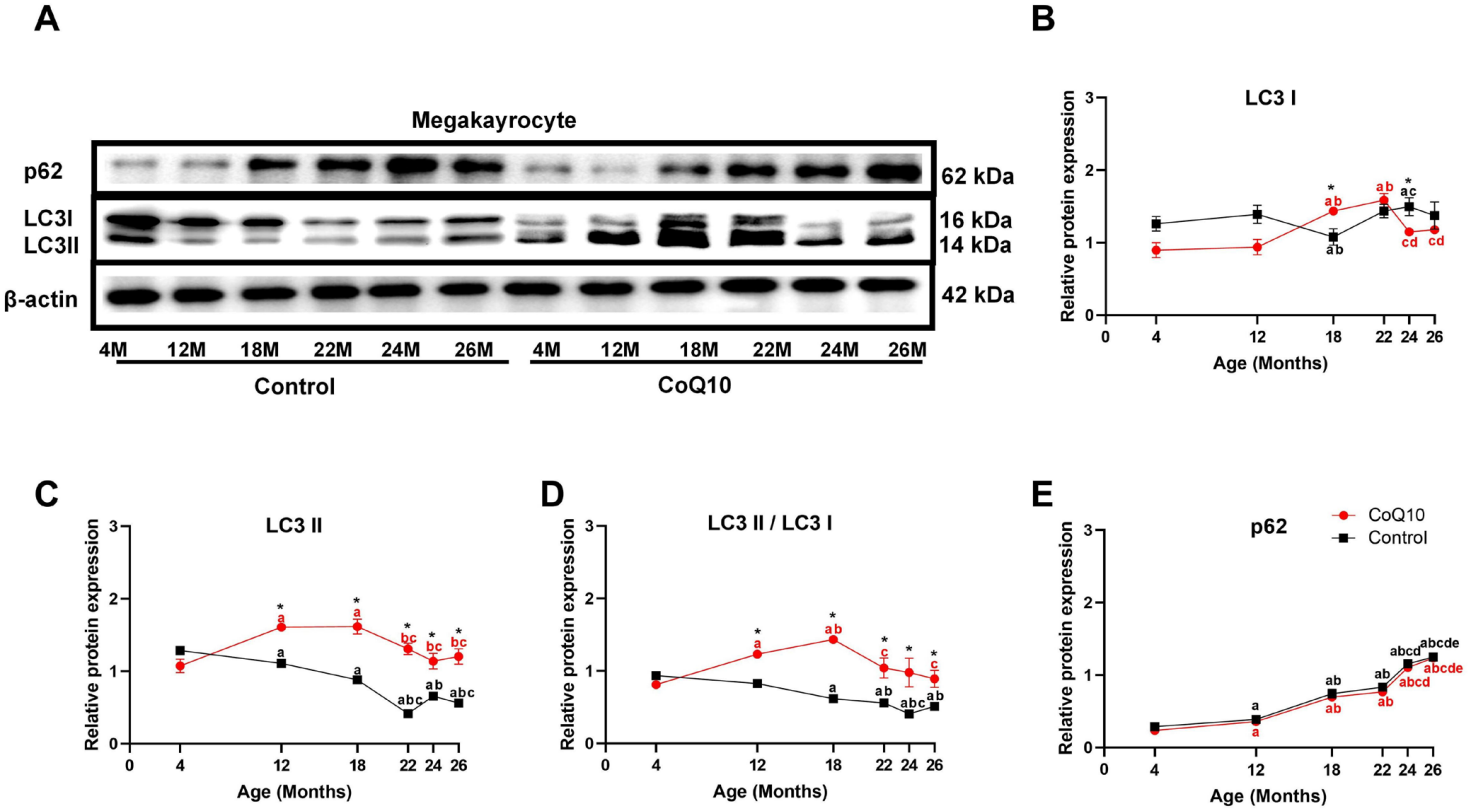
Coenzyme Q10 promotes autophagy activation in megakaryocytes during natural aging in mice. (A) Western blot detection of LC3I, LC3II, P62 protein levels in mouse bone marrow megakaryocytes, with β‐Actin as the internal control; (B–E) Density analysis using ImageJ software (*n* = 6). Data are presented as mean ± SEM. a: *p* < 0.05 compared with 4‐month‐old group (4 M); b: *p* < 0.05 compared with 12‐month‐old group (12 M); c: *p* < 0.05 compared with 18‐month‐old group (18 M); d: *p* < 0.05 compared with 22‐month‐old group (22 M); e: *p* < 0.05 compared with 24‐month‐old group (24 M); *:*p* < 0.05 Compared with the same age group.

To elucidate the mechanism by which CoQ10 promotes maturation and differentiation of bone marrow megakaryocytes in aged mice, proteomic analysis was conducted on MKs isolated from 24‐month‐old control and CoQ10‐treated mice. A total of 12 samples (6 per group) were analyzed. The complete proteomics results and the corresponding data annotations are provided in Supporting Information Table S1. Further inspection of the top five categories revealed significant upregulation of proteins involved in protein deacetylation in MKs from CoQ10‐treated mice (Figure 5A–C). KEGG pathway enrichment analysis indicated a significant upregulation of the proteasome pathway, along with pathways associated with cell growth and death (Figure 5D,E). Fisher's exact test was applied to identify the top 5 enriched biological process categories, which are presented in Figure 5C. The results showed that COPS3 protein expression was highest during protein dennedylation in biological processes. Notably, COPS3, a core component of the COP9 signalosome, was significantly upregulated following 12 weeks of CoQ10 supplementation. Age‐related analysis demonstrated a gradual decline in COPS3 expression with advancing age, whereas CoQ10 supplementation significantly restored its expression, particularly at 24 months (*p* < 0.05) (Figure 5F). In summary, these findings indicate that CoQ10 mitigates the age‐associated decline in COPS3 expression, enhances autophagy, promotes megakaryocyte maturation, and attenuates apoptosis in naturally aging mice.

**FIGURE 5:**
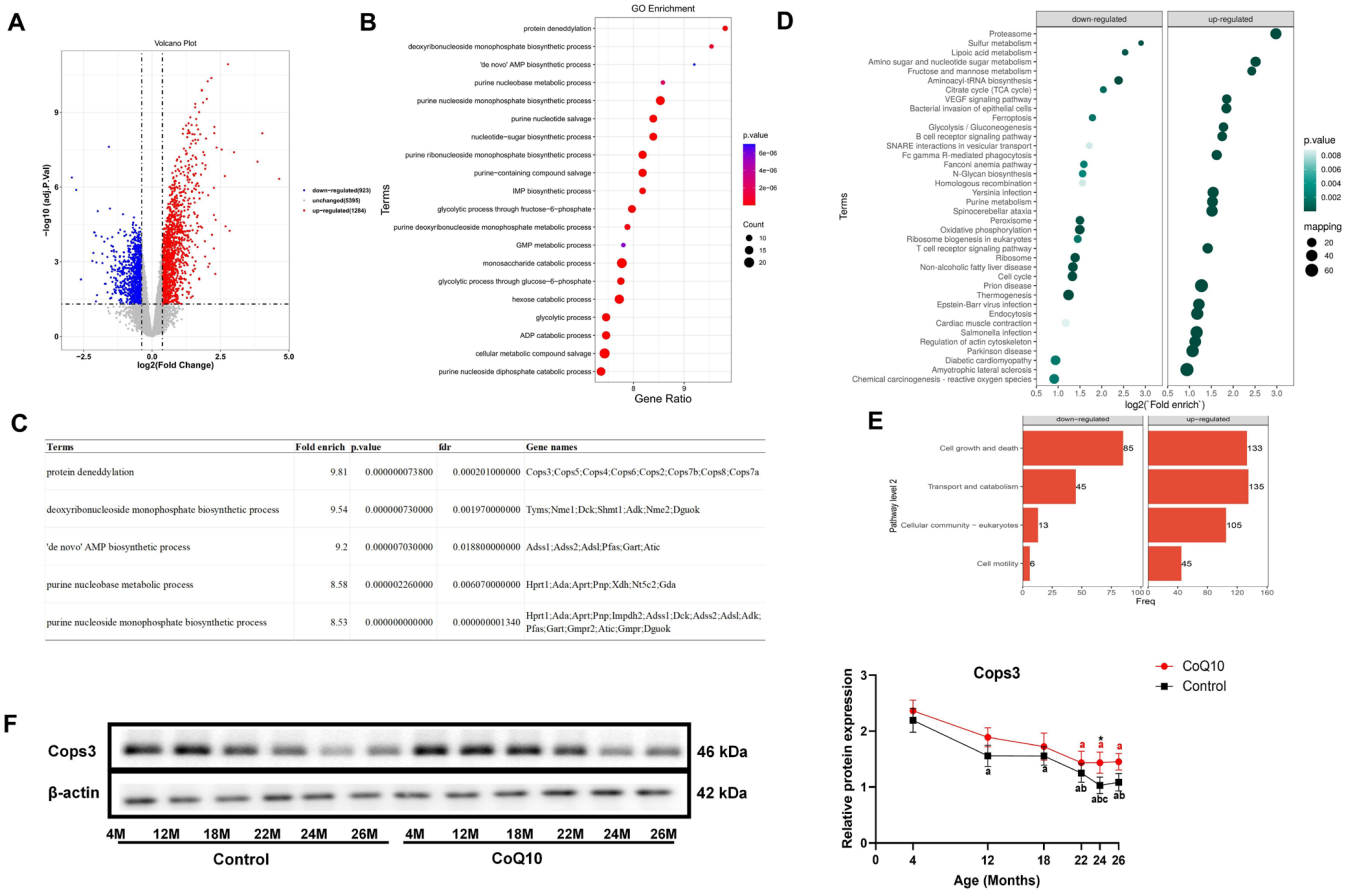
Proteomic identification and age‐dependent validation of CoQ10‐associated COPS3 upregulation in megakaryocytes. (A) Volcano plot showing differentially expressed proteins between CoQ10‐treated and control megakaryocytes. Proteins with FC > 1.3 or FC < 1/1.3 and a Benjamini‐Hochberg‐adjusted *p* < 0.05 were considered differentially expressed. Red, blue and gray points indicate upregulated, downregulated and unchanged proteins, respectively; (B) Gene Ontology annotations，Biological processes; (C) Go enrichment analysis of the top 5 biological processes and the associated proteins; (D) KEGG pathway enrichment; (E) KEGG level 2 functional classification of differentially expressed proteins; (F) Western blot detection of COPS3 protein levels in mouse bone marrow megakaryocytes, with β‐Actin as the internal control, Density analysis using ImageJ software (*n* = 6). Data are presented as mean ± SEM. a: *p* < 0.05 compared with 4‐month‐old group (4 M); b: *p* < 0.05 compared with 12‐month‐old group (12 M); c: *p* < 0.05 compared with 18‐month‐old group (18 M); *: *p* < 0.05 Compared with the same age group.

### 2.4 CoQ10 Upregulates COPS3 Expression in Senescent MEG‐01 Cells, Enhancing Autophagy, Maturation, and Differentiation

Dose–response analyses showed that 100 μM H_2_O_2_ markedly increased SA‐β‐gal positivity without reducing viability, whereas 100 μM CoQ10 did not affect viability; these concentrations were therefore selected for subsequent experiments (Figure S3). Senescence in MEG‐01 cells was induced using 100 μM H_2_O_2_. Autophagy was pharmacologically modulated with rapamycin (100 μM) as an inducer and LY294002 (100 μM) as an inhibitor. After 7 days of CoQ10 treatment, autophagy‐related protein expression was analyzed. Co‐treatment with rapamycin and CoQ10 markedly reduced p62 levels, whereas the LY294002‐treated group exhibited the highest p62 accumulation (Figure 6A,B). Analysis of COPS3 protein expression revealed that the combination of rapamycin and CoQ10 induced the strongest COPS3 upregulation, while CoQ10 treatment in the LY294002 group significantly elevated COPS3 levels compared with LY294002 alone (Figure 6C). Similarly, rapamycin combined with CoQ10 produced the highest LC3‐II expression and LC3‐II/LC3‐I ratio in MEG‐01 cells (Figure 6D–F). These findings demonstrate that CoQ10 promotes COPS3 expression and enhances autophagic activity in senescent megakaryocytes, even under conditions of autophagy inhibition. Notably, CoQ10 supplementation rescued the H_2_O_2_ induced autophagic impairment, supporting its role in restoring autophagic flux in aging megakaryocytes.

**FIGURE 6:**
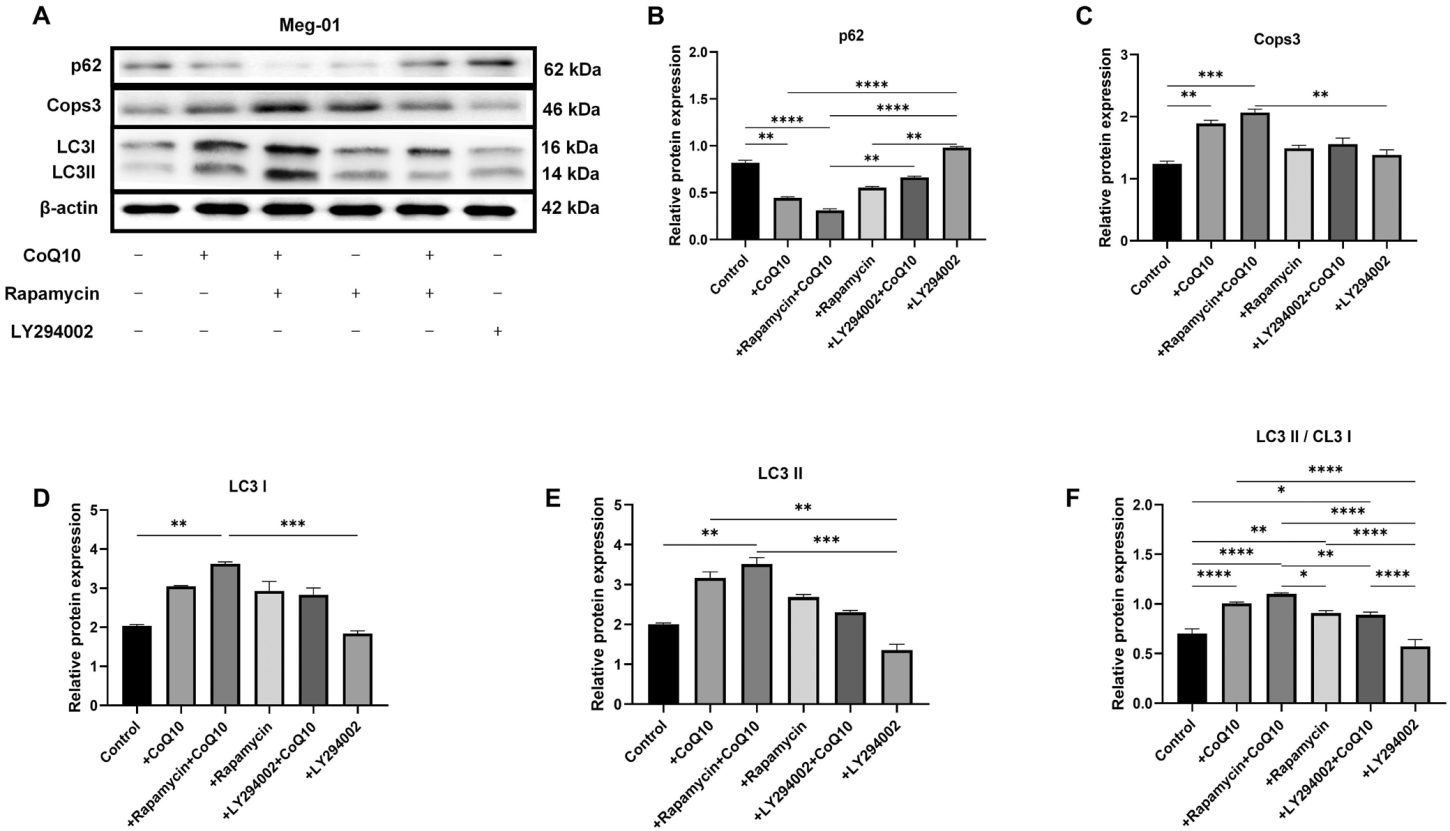
CoQ10 Enhances COPS3 expression and autophagy in senescent MEG‐01 cells. (A) Western blot detection of COPS3, LC3I, LC3II, P62 protein levels in MEG‐01 cell, with β‐Actin as the internal control; (B–F) Density analysis using ImageJ software (*n* = 6). Data are presented as mean ± SEM. *p < 0.05, **p < 0.01, ****p < 0.001, and *****p < 0.0001.

Flow cytometric analysis was performed to evaluate CD41^+^, CD61^+^, and CD41^+^CD61^+^ expression in MEG‐01 cells. The combined treatment with rapamycin and CoQ10 yielded the highest expression levels of all three markers, whereas LY294002 alone produced the lowest levels. Notably, compared with LY294002 treatment alone, the combination of CoQ10 and LY294002 significantly increased CD41^+^, CD61^+^, and CD41^+^CD61^+^ expression (*p* < 0.05), indicating that CoQ10 promotes the maturation and differentiation of senescent megakaryocytes even under conditions of autophagy inhibition (Figure 7A–C).

**FIGURE 7:**
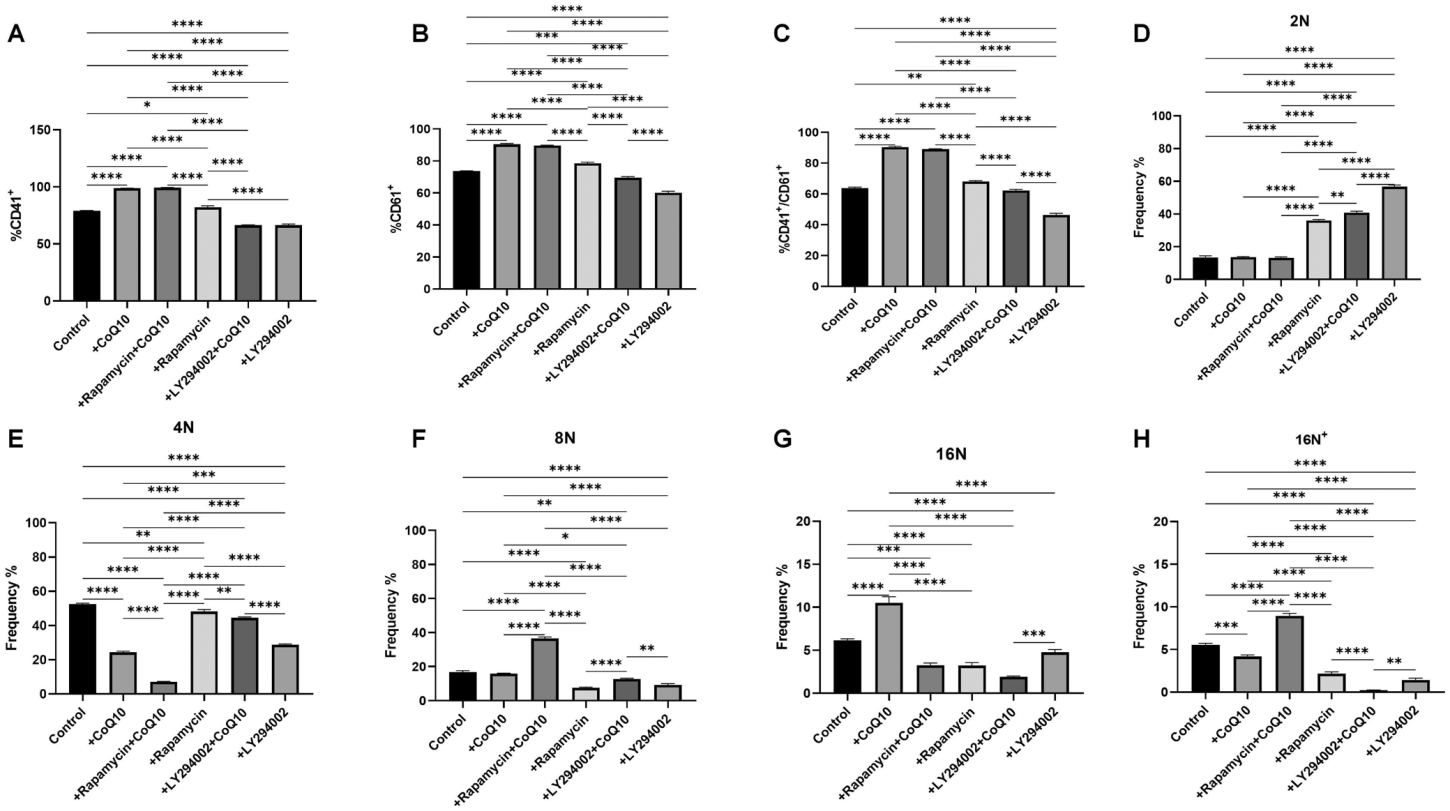
CoQ10 facilitates MEG‐01 maturation and differentiation during senescence. (A–C) Flow cytometry was employed to detect and quantify the expression of CD41, CD61, and CD41CD61 in the bone marrow of MEG‐01 cells; (D–H) The DNA ploidy distribution (the percentage of cells with 2n–16n+) was analyzed by flow cytometry (*n* = 6). Data are presented as mean ± SEM. *p < 0.05, **p < 0.01, ****p < 0.001, and *****p < 0.0001.

Polyploidy analysis further revealed that combined treatment with rapamycin and CoQ10 produced the highest proportions of 8 N, 16 N, and ≥ 16 N cells across all H_2_O_2_ concentrations, while LY294002 alone resulted in an accumulation of 2 N cells. CoQ10 co‐treatment with LY294002 significantly increased the proportions of 8 N and 16 N cells compared with LY294002 alone (*p* < 0.05) (Figure 7D–H).

### 2.5 COPS3 Contributes to CoQ10‐Mediated Autophagy Regulation and Functional Improvement in Senescent MEG‐01 Cells

To determine whether COPS3 contributes to the effects of CoQ10, we performed COPS3 loss‐of‐function experiments in senescent MEG‐01 cells using siRNA. qRT‐PCR and Western blotting confirmed that COPS3 expression was markedly reduced following COPS3 siRNA transfection compared with non‐targeting siRNA transfection (*p* < 0.05) (Figure 8A–C). In senescent cells transfected with non‐targeting siRNA, CoQ10 treatment increased the LC3‐II/LC3‐I ratio and decreased p62 expression, consistent with enhanced autophagy. These CoQ10‐induced changes were attenuated following COPS3 knockdown (*p* < 0.05) (Figure 8D,E). Similarly, COPS3 silencing partially reversed the CoQ10‐induced changes in megakaryocyte maturation and differentiation (*p* < 0.05) (Figure 8F,G).

**FIGURE 8:**
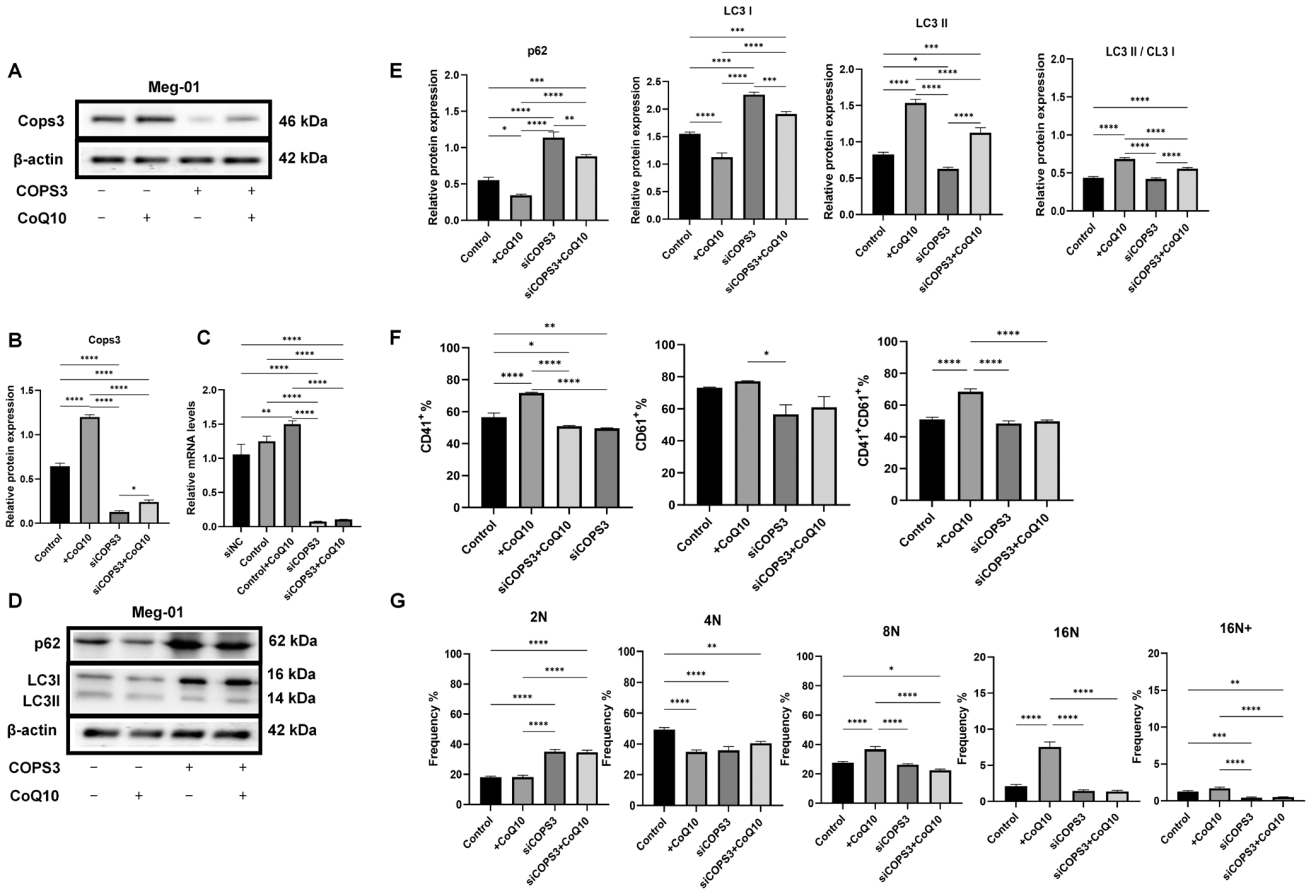
Effects of COPS3 knockdown on CoQ10‐associated autophagy regulation and megakaryocyte phenotypes. (A) Western blot detection of COPS3 protein levels in MEG‐01 cell, with β‐Actin as the internal control; (B) Density analysis using ImageJ software; (C) Qrt‐PCR analysis of MEG‐01 cells transfected with COPS3 siRNA; (D) Western blot detection of P62, LC3I, LC3II protein levels in MEG‐01 cell, with β‐Actin as the internal control; (E) Density analysis using ImageJ software; (F) Flow cytometry was employed to detect and quantify the expression of CD41, CD61 and CD41CD61 in the bone marrow of MEG‐01 cells; (G) The DNA ploidy distribution (the percentage of cells with 2n–16n+) was analyzed by flow cytometry (*n* = 6). Data are presented as mean ± SEM. *p < 0.05, **p < 0.01, ****p < 0.001, and *****p < 0.0001.

### 2.6 CoQ10 Enhances Autophagic Flux in Senescent MEG‐01 Cells

To determine whether the CoQ10‐associated changes in LC3‐II and p62 reflected enhanced autophagic flux rather than impaired autophagic degradation, we performed an LC3‐II turnover assay in senescent MEG‐01 cells using Bafilomycin A1. Cells were treated with vehicle or CoQ10 in the presence or absence of Bafilomycin A1. Bafilomycin A1 increased LC3‐II accumulation in both vehicle‐ and CoQ10‐treated cells, consistent with the inhibition of lysosomal degradation. Notably, LC3‐II accumulation was greater in the CoQ10 plus Bafilomycin A1 group than in the Bafilomycin A1‐only group. Accordingly, the Bafilomycin A1‐sensitive increase in LC3‐II was more pronounced following CoQ10 treatment. CoQ10 also decreased p62 abundance under lysosome‐competent conditions, whereas Bafilomycin A1 attenuated this reduction (*p* < 0.05) (Figure 9A–F). Similarly, treatment with Bafilomycin A1 attenuated the maturation and differentiation of MEG‐01 cells (*p* < 0.05) (Figure 9G,H).

**FIGURE 9:**
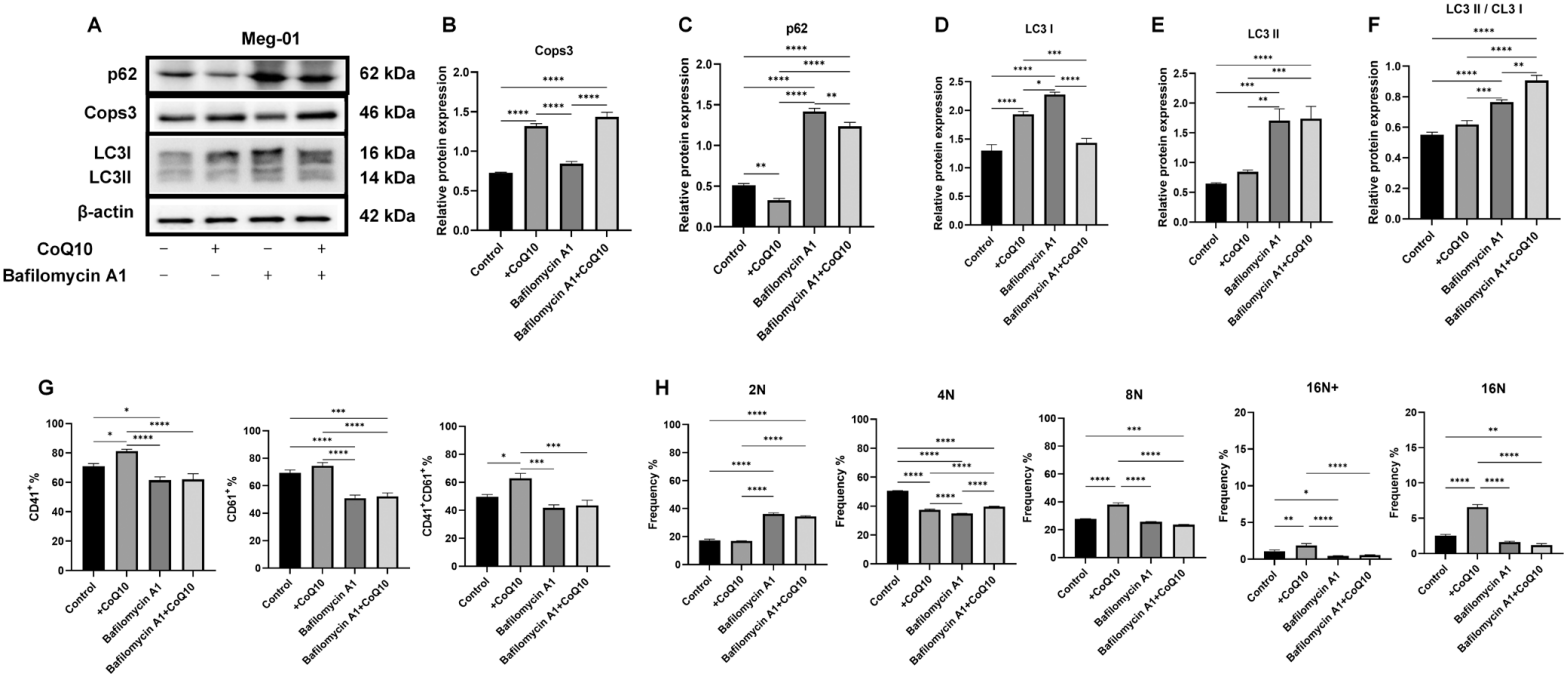
CoQ10 Enhances autophagic flux in senescent MEG‐01 cells. (A) Western blot detection of COPS3, P62, LC3I, LC3II protein levels in MEG‐01 cell, with β‐Actin as the internal control; (B–F) Density analysis using ImageJ software; (G) Flow cytometry was employed to detect and quantify the expression of CD41, CD61 and CD41CD61 in the bone marrow of MEG‐01 cells; (H) The DNA ploidy distribution (the percentage of cells with 2n–16n+) was analyzed by flow cytometry (*n* = 6). Data are presented as mean ± SEM. *p < 0.05, **p < 0.01, ****p < 0.001, and *****p < 0.0001.

### 2.7 Dietary CoQ10 Improves Platelet Aggregation and Activation in Naturally Aged Mice

Platelet function analysis revealed that collagen‐induced aggregation progressively increased with age, peaking before declining at 26 months. CoQ10 supplementation significantly suppressed platelet aggregation in mice aged 18, 22, 24, and 26 months (*p* < 0.05). Similarly, platelet activation markers CD62P and JON/A showed age‐dependent elevations followed by a decline at 26 months. CoQ10 administration markedly reduced CD62P expression at 22, 24, and 26 months and attenuated platelet activation across the 18–26 month range (*p* < 0.05) (Figure 10A–C). In naturally aging mice, both platelet and reticulated platelet counts increased with age, reaching their highest levels at 24 months; however, dietary CoQ10 intervention did not affect either count(Figure 10D,E). The duration of tail bleeding extended with age and then shortened at 22 months, yet CoQ10 treatment did not significantly influence bleeding time (Figure 10E).

**FIGURE 10:**
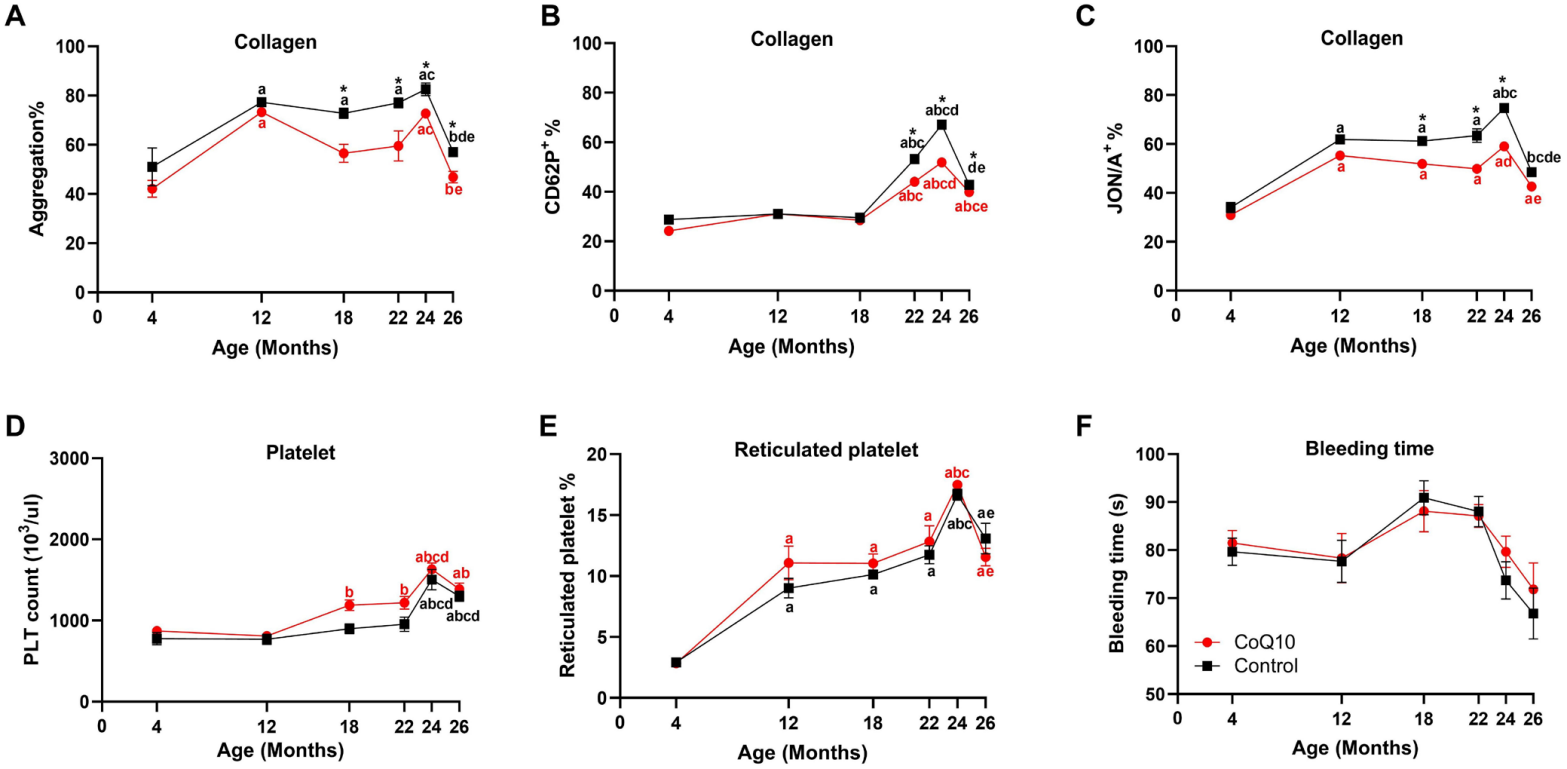
CoQ10 attenuates platelet function in naturally aged mice. (A) Collagen stimulates platelet aggregation rate; (B) Rate of collagen‐stimulated platelet CD62P expression; (C) Rate of collagen‐stimulated platelet JON/A expression; (D) Platelet count; (E) Reticulate Platelet percentage; (F) Bleeding time (*n* = 7). Data are presented as mean ± SEM. a: *p* < 0.05 compared with 4‐month‐old group (4 M); b: *p* < 0.05 compared with 12‐month‐old group (12 M); c: *p* < 0.05 compared with 18‐month‐old group (18 M); d: *p* < 0.05 compared with 22‐month‐old group (22 M); e: *p* < 0.05 compared with 24‐month‐old group (24 M); *: *p* < 0.05 Compared with the same age group.

## 3 Discussion

Our findings indicate that dietary CoQ10 alleviates age‐related megakaryocyte dysfunction in naturally aged mice. CoQ10 improved systemic redox and inflammatory profiles, enhanced MK proliferation, maturation, and polyploidization, and reduced platelet hyperreactivity without altering platelet counts. Mechanistically, the combined pharmacological, proteomic, autophagic‐flux, and COPS3‐knockdown results support a model in which COPS3 contributes to CoQ10‐associated autophagy regulation and improved MK function. These data identify COPS3 as a functional contributor rather than the sole or sufficient mediator of CoQ10 action.

Aged mice exhibited increased oxidative stress, characterized by elevated ROS and MDA levels and reduced total antioxidant capacity and antioxidant enzyme activities. CoQ10 supplementation attenuated these alterations, consistent with its established roles in mitochondrial electron transport and antioxidant defense (Noh et al. 2013; Hargreaves 2021; Sun et al. 2019; Jing et al. 2015). CoQ10 also reduced circulating IL‐1β, IL‐6 and TNF‐α, indicating an improvement in the chronic inflammatory state associated with aging. Because oxidative stress can promote NF‐κB‐dependent inflammatory signaling, these antioxidant and anti‐inflammatory effects may be functionally related (Ferrucci and Fabbri 2018; Abd El‐Aal et al. 2017).

The systemic response to CoQ10 was age‐dependent. Circulating ubiquinol and ubiquinone concentrations increased most clearly in mice older than 22 months, whereas younger cohorts showed limited changes (Figure S2). This pattern may reflect age‐related reductions in endogenous CoQ10 availability, mitochondrial function, or metabolic responsiveness. It may also indicate that the biological effects of supplementation become more evident during advanced aging (Barcelos and Haas 2019; Garrido‐Maraver et al. 2014; Sharma et al. 2016; Cirilli et al. 2024; Schniertshauer et al. 2018).

At the cellular level, CoQ10 improved several features of bone marrow MK function. MK differentiation and platelet production are metabolically demanding processes and may be particularly sensitive to mitochondrial dysfunction during aging (de Jonckheere et al. 2023; Xu et al. 2025). CoQ10 increased CFU‐Meg formation, maturation‐marker expression, and the proportion of highly polyploid MKs while reducing apoptosis (Chen et al. 2013; Xu et al. 2025). These findings are consistent with improved cellular homeostasis in aged MKs, although the relative contributions of mitochondrial metabolism, redox regulation, and other pathways remain to be defined.

Autophagy is required for the removal of damaged cellular components and declines during aging, contributing to hematopoietic dysfunction (Zhao et al. 2025; Cuervo 2008; Gómez‐Virgilio et al. 2022). In bone marrow MKs, aging was accompanied by a reduced LC3‐II/LC3‐I ratio and increased p62 abundance, whereas CoQ10 shifted these markers in the opposite direction. Because LC3‐II and p62 were measured under steady‐state conditions in the animal experiments, these changes are consistent with altered autophagic activity but do not independently establish enhanced autophagic flux in vivo.

Pharmacological modulation with rapamycin and LY294002 further linked the MK phenotypes to autophagy‐associated signaling. Rapamycin produced changes consistent with autophagy activation, whereas LY294002 attenuated the effects of CoQ10. However, because these compounds affect signaling pathways beyond autophagy, the pharmacological findings were interpreted together with the newly added genetic and flux experiments rather than as standalone evidence of mechanism.

Proteomic analysis identified coordinated changes in COP9 signalosome components following CoQ10 treatment. COPS2, COPS3, COPS4, COPS5, COPS6, COPS7A, COPS7B, and COPS8 were increased, with COPS3 showing the largest effect size among the reliably quantified subunits. Western blotting across age groups further supported increased COPS3 protein abundance following CoQ10 treatment. The COP9 signalosome regulates protein stability through deneddylation and modulation of Cullin‐RING ubiquitin ligases (Chamovitz 2009; Qin et al. 2020; Suisse et al. 2018). These functions provide a plausible connection between COPS3, proteostasis, and autophagy‐associated regulation (Zhang et al. 2023, 2018; Niu et al. 2023). Nevertheless, the concurrent alteration of several COP9 subunits indicates that the response is not restricted to COPS3.

The siRNA experiments provide direct loss‐of‐function evidence for the involvement of COPS3. In senescent MEG‐01 cells, COPS3 knockdown attenuated the CoQ10‐associated increase in the LC3‐II/LC3‐I ratio and reduction in p62 abundance. It also partially diminished the effects of CoQ10 on MK maturation‐marker expression and polyploidization. The incomplete attenuation suggests that COPS3 contributes to these effects but is unlikely to be their only mediator. Because gain‐of‐function and rescue experiments were not performed, the present data do not establish that COPS3 is sufficient for CoQ10 action.

The Bafilomycin A1 experiments provide additional evidence that CoQ10 enhances autophagic flux in senescent MEG‐01 cells. LC3‐II accumulation was greater following combined CoQ10 and Bafilomycin A1 treatment than following Bafilomycin A1 alone, indicating increased delivery of LC3‐II to the lysosomal degradation pathway. CoQ10 also reduced p62 under lysosome‐competent conditions, and this change was attenuated by Bafilomycin A1. Because p62 can also be regulated through autophagy‐independent mechanisms, LC3‐II turnover was considered the primary flux measurement. These findings support enhanced autophagic flux in senescent MEG‐01 cells rather than simple steady‐state autophagosome accumulation.

CoQ10 is primarily recognized as a mitochondrial electron carrier and lipid‐soluble antioxidant, but it can also influence nuclear gene‐expression programs related to metabolism, inflammation, and stress responses. Improved mitochondrial electron transport, ATP availability, and redox balance could therefore affect nuclear‐encoded proteins involved in proteostasis (Mantle and Golomb 2025; Hidalgo‐Gutiérrez et al. 2021; Hargreaves 2014). Although CoQ10 is primarily recognized as a mitochondrial electron carrier and lipid‐soluble antioxidant, previous studies indicate that it can also influence nuclear gene‐expression programs. Gene‐expression profiling in human CaCo2 cells showed that CoQ10 altered genes involved in cellular signaling, metabolism, transport, and transcriptional regulation (Pallotti et al. 2021; Groneberg et al. 2005; Fischer et al. 2011). In animal models, CoQ10 modulated the redox‐sensitive Nrf2 and NF‐κB pathways and increased nuclear PGC‐1α together with mitochondrial protein expression (Bagheri et al. 2025; Pala et al. 2016; Hocum Stone et al. 2019). These findings suggest that changes in mitochondrial and redox homeostasis may be transmitted to nuclear transcriptional programs. The increased COPS3 expression observed in our study may therefore represent an indirect response to CoQ10‐associated redox or mitochondrial signaling. However, no direct CoQ10‐responsive transcriptional regulator of COPS3 has been identified, and the present study did not examine COPS3 promoter activity, transcription‐factor occupancy, or mRNA stability. Thus, the upstream mechanism linking CoQ10 to increased COPS3 expression remains unresolved (Pala et al. 2016; Hocum Stone et al. 2019).

The reduced platelet aggregation and activation observed in aged mice is broadly consistent with previous evidence that CoQ10 can modulate human platelet and vascular function (Serebruany et al. 1997; Ya et al. 2019; Wang et al. 2022). In a small supplementation study, CoQ10 increased circulating CoQ10 concentrations and altered platelet size and selected surface receptors. Studies using isolated human platelets have also reported reduced aggregation, secretion, integrin activation, and spreading following CoQ10 exposure. These effects have been linked to enhanced cAMP—protein kinase A signaling and reduced oxidative stress (Ya et al. 2019). However, these studies were small or conducted ex vivo and did not assess clinical thrombotic outcomes. They therefore support the biological plausibility of our findings but do not establish that CoQ10 prevents thrombosis in older adults. In the present study, the reductions in platelet aggregation and activation were not accompanied by changes in platelet count or prolongation of tail bleeding time. Thus, CoQ10 did not produce a detectable impairment of primary hemostasis under the experimental conditions examined. However, tail bleeding time provides only a limited assessment of hemostatic function and does not evaluate coagulation, bleeding under pathological challenge, or interactions with anticoagulant or antiplatelet therapies. Therefore, these findings should not be interpreted as establishing a favorable hemostatic safety profile, particularly in elderly or comorbid populations.

Broader clinical evidence provides indirect support for the cardiovascular relevance of CoQ10. In the Q‐SYMBIO trial, adjunctive CoQ10 treatment in patients with chronic heart failure was associated with fewer major adverse cardiovascular events and improved clinical outcomes (Mortensen et al. 2014). Other randomized studies and meta‐analyses have reported improvements in endothelial function in cardiovascular or metabolic disorders. Combined selenium and CoQ10 supplementation has also been associated with reduced cardiovascular mortality in community‐dwelling older adults (Alehagen et al. 2013; Hernández‐Camacho et al. 2018; Mantle et al. 2022; Díaz‐Casado et al. 2019; Ayunin et al. 2022). However, these populations and outcomes differ substantially from the present aging model. Moreover, the combined supplementation design does not isolate the contribution of CoQ10. Thus, the available clinical evidence supports further investigation but cannot be extrapolated to the prevention of platelet‐mediated thrombosis during physiological aging.

The use of naturally aged mice was important for evaluating CoQ10 under conditions of chronological aging. Unlike accelerated genetic aging models, naturally aged mice develop cumulative mitochondrial, metabolic, inflammatory, and hematopoietic alterations without a predefined mutation driving the phenotype. This model is therefore well suited for assessing a long‐term dietary intervention across the lifespan. However, natural aging requires prolonged observation and is accompanied by greater biological heterogeneity, age‐associated comorbidities, and potential survival selection. In contrast, accelerated aging models, such as Klotho‐deficient and BubR1 hypomorphic mice, develop earlier and more reproducible phenotypes and are valuable for examining specific aging‐related pathways (Fischer et al. 2010; Bian et al. 2015; Pun et al. 2025). Nevertheless, their phenotypes arise from defined genetic defects and may include developmental or tissue‐specific abnormalities that are not representative of physiological aging. Thus, naturally aged and genetic aging models provide complementary information, and future studies using pathway‐specific models may help clarify the mechanisms underlying the effects of CoQ10 on megakaryocyte aging.

Several limitations of this study should be considered when interpreting these findings. First, the mechanistic experiments used an H_2_O_2_‐induced senescence model in MEG‐01 cells. Autophagic flux was not directly assessed in primary MKs or aged mice. Moreover, the in vitro CoQ10 concentration of 100 μM exceeds the circulating concentrations typically achieved after oral supplementation. It should therefore be interpreted as a pharmacological exposure for mechanistic studies rather than as a physiologically relevant concentration. The limited aqueous solubility and cellular uptake of CoQ10 complicate extrapolation from nominal culture concentrations to systemic exposure. Nevertheless, the consistent phenotypic changes observed in the dietary mouse model support the biological relevance of the in vitro findings without establishing dose or pharmacokinetic equivalence. In addition, only one dietary concentration of CoQ10 (550 mg/kg chow) was evaluated. This design precluded determination of dose–response relationships, the minimum effective dose, and the optimal dosing range. Second, COPS3 knockdown provided loss‐of‐function evidence supporting the involvement of COPS3. Gain‐of‐function studies are needed to determine whether COPS3 is sufficient to produce the observed effects. Rescue experiments would further strengthen the causal interpretation of the knockdown results. Third, only male mice were included in this study. Sex differences in aging, CoQ10 metabolism, MK biology, and platelet reactivity may influence responses to CoQ10. Therefore, these findings should not be directly generalized to female mice. Studies incorporating age‐matched female cohorts are needed to determine whether the effects of CoQ10 on COPS3‐associated autophagy, MK function, and platelet reactivity are sex dependent. Finally, the findings were obtained in naturally aged mice. Whether they extend to human megakaryopoiesis and thrombotic risk requires validation in clinical studies. These limitations constrain the strength and translational scope of our mechanistic conclusions. However, they do not alter the observed associations among CoQ10 treatment, COPS3 expression, autophagy‐related markers, and MK function.

In conclusion, dietary CoQ10 alleviated age‐related MK dysfunction and reduced platelet hyperreactivity in naturally aged mice. The pharmacological experiments, COPS3 knockdown and Bafilomycin A1 flux assay collectively indicate that COPS3‐associated autophagy contributes to these effects. These findings support further investigation of CoQ10 as a nutritional strategy for age‐related platelet dysfunction while avoiding conclusions regarding clinical thrombosis prevention.

## 4 Materials and Methods

### 4.1 Animals

C57BL/6J male mice were purchased from BESTEST (Zhuhai BesTest Bio‐Tech). A total of 84 mice were stratified into six pretreatment age cohorts of 1, 9, 15, 19, 21, and 23 months, with 14 mice per cohort. Within each age cohort, mice were randomly allocated in a 1:1 ratio to receive either the control diet or the same diet supplemented with CoQ10 (Aladdin) at 550 mg per kg of diet, resulting in seven mice per age‐by‐diet group. Based on the recorded food intake and body weight, the daily CoQ10 exposure was calculated as dietary CoQ10 concentration (0.55 mg/g) × daily food intake (g/day)/body weight (kg), and the human‐equivalent dose (HED) was subsequently calculated using body‐surface‐area normalization as HED (mg/kg/day) = mouse dose (mg/kg/day) × 3/37, corresponding to approximately 300 mg/day for a 60‐kg adult (Reagan‐Shaw et al. 2008). After 12 weeks of intervention, mice were analyzed at 4, 12, 18, 22, 24, and 26 months of age. At the end of the 12‐week intervention, the CoQ10‐containing diet was withdrawn, and blood samples were collected 12 h later at a consistent time point for all animals. Mice were housed in a specific pathogen‐free (SPF) facility under controlled environmental conditions: 25°C ± 2°C, 50% ± 5% relative humidity, and a 12‐h light/dark cycle. Food intake and body weight were recorded weekly. All animal procedures were approved by the Laboratory Animal Ethics Committee of the Sun Yat‐sen University Health System (approval no. SYSU‐IACUC‐2021‐00471) and conducted in accordance with the Guide for the Care and Use of Laboratory Animals. Seven mice were assigned to each age‐by‐diet group to provide at least six evaluable biological replicates for assays with greater sample‐quality or material requirements.

Each mouse constituted one independent biological replicate. Samples from individual mice were collected and processed separately and were not pooled. Where technical replicate measurements were performed, their mean was calculated for each mouse and treated as one biological observation in the statistical analyses. The final sample size was seven mice per age‐by‐diet group for circulating biomarker and platelet‐function analyses. Selected analyses included six mice per age‐by‐diet group because one sample per group yielded insufficient viable cells or failed to meet predefined quality‐control criteria. The exact number of biological replicates included in each analysis is provided in the corresponding figure legend and animal‐allocation flowchart. Individual cells, colonies, microscopic fields, and technical replicate wells were not treated as independent biological replicates (Figure S4).

### 4.2 Megakaryocyte Isolation and Culture

Megakaryocytes were isolated and cultured as previously described. Bone marrow was flushed from the femur and tibia using Hank's Balanced Salt Solution (HBSS) supplemented with 5% fetal bovine serum (FBS). After dissociation, cells were cultured in Iscove's Modified Dulbecco's Medium (IMDM) containing L‐glutamine, 25 mM HEPES, and 5% FBS (Kimmerlin et al. 2021).

### 4.3 Blood Counts

Blood samples were collected from the hearts of mice into anticoagulant tubes containing ACD (Yuanye biotechnology). Complete blood cell counts were measured using a standard hematology analyzer.

### 4.4 Megakaryocyte Cell Line Culture

Megakaryocyte cell lines MEG‐01 cells were purchased from ATCC (American Type Culture Collection). Cells were cultured in RPMI 1640 medium supplemented with 10% fetal bovine serum in a 37°C, 5% CO_2_ incubator. Human thrombopoietin (200 ng/mL, PeproTech) was added to each group of cells and induced for 7 days of differentiation. The medium was changed midway through the process, and thrombopoietin at the same concentration was added. Further testing was performed after 7 days of induction. MEG‐01 cells were induced to undergo senescence by treatment with H_2_O_2_ at concentrations of 100 μM. Rapamycin (100 μM, Sigma), LY294002 (100 μM, Sigma), and Bafilomycin A1 (100 nM, Sigma) were used. MEG‐01 cells were treated with CoQ10 (100 μM, Sigma) as described previously (Wang et al. 2022; Ya et al. 2020). Senescent MEG‐01 cells were transfected with COPS3‐targeting siRNA or non‐targeting control siRNA at a final concentration of 20 nM. After 48 h, cells were treated with or without CoQ10, and COPS3 knockdown and associated changes in LC3‐I/II, p62, CD41/CD61 expression, and DNA ploidy were assessed by qRT‐PCR, Western blotting, and flow cytometry, as appropriate. Rapamycin, LY294002, CoQ10, and Bafilomycin A1 were dissolved in DMSO, and the final DMSO concentration was matched across all treatment and vehicle‐control groups, including combination‐treatment conditions.

### 4.5 Mouse Platelet Isolation, Aggregation, and Activation Assays

Washed mouse platelets were prepared as previously described (Ya et al. 2020). Briefly, fresh blood was drawn from mouse hearts and anti‐coagulated with ACD (Yuanye biotechnology Company). Platelets were isolated by differential centrifugation of whole blood and washed twice with PIPES buffer. Platelet aggregation was measured in a turbidometric platelet aggregometer at 37°C with stirring (1000 rpm). Washed platelets (3 × 10^8^/ml) in modified PIPES buffer were stimulated with Collagen.

### 4.6 Flow Cytometric Analysis

Bone marrow‐derived megakaryocytes were stained with biotin‐labeled anti‐mouse lineage antibody, followed by staining with fluorescein‐conjugated anti‐mouse surface antibodies (FITC‐CD41, PE‐CD61, BD). Cells were fixed with 70% ethanol overnight at 4°C, washed, and incubated with DAPI/Triton X‐100 solution (0.1% Triton X‐100, 1 μg/mL DAPI, Beyotime) (Muntean et al. 2007). Apoptosis was assessed using Annexin V‐APC staining (eBioscience). For reticulated platelet analysis, whole blood was incubated with thiazole orange (Sigma) dye at room temperature for 2.5 h. Washed platelets (2.5 × 10^5^/μl) were diluted in modified PIPES buffer, and activated by the addition of calcium (1 mM), and stimulated with Collagen (Chrono‐Log). At 2 min after activation, CD62P‐APC (BD) and JON/A‐PE (emfret) were added. After 20 min, platelets were fixated by adding 1:4 cold PFA 4%. Samples were analyzed using a CytoFLEX S flow cytometer, and data were processed with CytExpert 2.4 software (Kienast and Schmitz 1990).

### 4.7 Megakaryocytes Colony‐Forming Unit Assay

Megakaryocytes were seeded in methylcellulose medium (STEMCELL Technologies) and incubated for 7 days. Colonies were stained with acetylcholinesterase (AchE, MBL) and counted. For proplatelet formation, megakaryocytes were plated on fibrinogen‐coated (100 μg/mL) culture dishes and incubated at 37°C for 3 h. Proplatelet formation was visualized using a Carl Zeiss Axio Vert.A1 microscope (200 × magnification).

### 4.8 Enzyme‐Linked Immunosorbent Assay (ELISA)

ELISA was performed according to the manufacturer's instructions. The plasma samples extracted from mice of different ages were analyzed to quantify the levels of IL‐6, IL‐1β, TNF‐α, IL‐10 (Biolegend).

### 4.9 Biochemical Assays

Total antioxidant capacity (T‐AOC), catalase (CAT), malondialdehyde (MDA), superoxide dismutase (SOD), and glutathione (GSH) were measured using commercial assay kits (Nanjing Jiancheng Bioengineering Institute).

### 4.10 Western Blotting

Bone marrow‐derived megakaryocytes and MEG‐01, Dami cells were lysed with RIPA buffer (Beyotime) containing protease and phosphatase inhibitors. Equivalent quantities of protein samples were subjected to SDS‐PAGE, after which they were transferred onto PVDC membranes. Subsequently, the membranes were blocked with 5% skimmed BSA and incubated overnight at 4°C with respective primary antibodies as follows, following incubation with HRP‐conjugated secondary antibodies (LC3I/II 1:1000, CST; p62, 1:1000, CST, COPS3,1:1000, CST and β‐actin, 1:2500, CST). Protein bands were detected using a Mini Chemiluminescent/Fluorescent Imaging and Analysis System and quantified by optical density analysis using ImageJ software.

### 4.11 Astral DIA Quantitative Proteomics Assay

Bone marrow megakaryocytes from 4, 24‐month‐old mice were collected and rapidly frozen. The samples were treated with lysis buffer containing multiple inhibitors to extract proteins, and the supernatant was collected via centrifugation. Protein concentration was determined using the BCA method. Equal amounts of protein were reduced, alkylated, and enzymatically digested into peptides. Mass spectrometry analysis was performed using the Astral DIA mode, and the data were processed by software for quantitative proteomics analysis. Differential protein abundance between the CoQ10‐treated and control groups was evaluated using a two‐sided Welch's *t*‐test applied to normalized log2‐transformed protein abundance values. *p*‐values were adjusted for multiple comparisons using the Benjamini‐Hochberg procedure. Proteins with a fold change greater than 1.3 or less than 1/1.3 and a Benjamini‐Hochberg‐adjusted *p*‐value below 0.05 were considered differentially expressed.

### 4.12 Statistical Analysis

Data are presented as standard error of the mean (SEM). Statistical analysis was performed using GraphPad Prism (version 8.0). Two‐way ANOVA followed by Dunnett's post hoc test was performed for within‐group comparisons. Correlation analyses were performed using Pearson's correlation coefficient. Statistical significance was defined as *p* < 0.05.

## Author Contributions

Conceptualization: Yixuan Xu, Yu‐heng Mao, Shuangfeng Xie, Fenglin Song, Fang He, CaiXia Wang, Yueying Wu, Minghan Wang, Heyu Ni, and Yan Yang; Data curation: Yixuan Xu, Fang He, CaiXia Wang, and Yueying Wu; Formal analysis: Yixuan Xu, Yu‐heng Mao, Shuangfeng Xie, Fenglin Song, Fang He, CaiXia Wang, Yueying Wu, Minghan Wang, Heyu Ni, and Yan Yang; Funding acquisition: Yan Yang; Investigation: Yixuan Xu, Shuangfeng Xie, Fenglin Song, Yu‐heng Mao, Fang He, CaiXia Wang, Yueying Wu, Heyu Ni, and Yan Yang; Methodology: Yixuan Xu, Shuangfeng Xie, Fenglin Song, Yu‐heng Mao, Fang He, CaiXia Wang, Yueying Wu, Heyu Ni, and Yan Yang; Project administration and supervision: Heyu Ni and Yan Yang; Writing: Yixuan Xu and Yan Yang; Review and editing: Yixuan Xu, Shuangfeng Xie, Fenglin Song, Yu‐heng Mao, Fang He, CaiXia Wang, Yueying Wu, Heyu Ni, and Yan Yang. All authors have read and agreed to the published version of the manuscript.

## Funding

This work was also supported by the Key Program of the National Natural Science Foundation of China (Grant nos. 82030098), the National Natural Science Foundation of China (Grant no. 82574083), the 2025 Annual Higher Education Research Project of the 14th Five‐Year Plan of Guangdong Province (25GZD003), and the 2025 Annual Project for Teaching Quality and Teaching Reform of Undergraduate Universities in Guangdong Province.

## Ethics Statement

Protocols for animal experiments were approved by the Sun Yat‐sen University Health System (SYSU‐IACUC‐2021‐00471) Laboratory Animal Ethics Committee and conducted in accordance with the Guide for the Care and Use of Laboratory Animals (National Institutes of Health, NIH).

## Conflicts of Interest

The authors declare no conflicts of interest.

## Supporting information


**Figure S1:** Coenzyme Q10 reduces oxidative stress levels in naturally aging mice. Plasma (A): MDA; (B): SOD; (C): CAT; (D): GSF level (*n* = 7). Data are presented as mean ± SEM. a: *p* < 0.05 compared with 4‐month‐old group (4 M); b: *p* < 0.05 compared with 12‐month‐old group (12 M); c: *p* < 0.05 compared with 18‐month‐old group (18 M); *: *p* < 0.05 Compared with the same age group.
**Figure S2:** Coenzyme Q10 supplementation increases ubiquinol and ubiquinone reduced CoQ10 levels in naturally aged mice Plasma samples used for ubiquinol and ubiquinone measurements were collected 12 h after withdrawal of the CoQ10‐containing diet. (A): ubiquinol; (B): ubiquinone level (*n* = 6). Data are presented as mean ± SEM. a: *p* < 0.05 compared with 4‐month‐old group (4 M)*: *p* < 0.05 Compared with the same age group.
**Figure S3:** Dose–response validation for the selection of H_2_O_2_ and CoQ10 concentrations in MEG‐01 cells. (A–D) Representative bright‐field images of SA‐β‐gal staining in MEG‐01 cells treated with 0, 1, 10 or 100 μM H_2_O_2_ for 7 day. Insets show magnified views of the boxed regions. Scale bars, 50 μm. (E) Quantification of SA‐β‐gal‐positive cells following H_2_O_2_ treatment. (F) Cell viability following treatment with 0, 1, 10, 100, 200 or 500 μM H_2_O_2_. (G) Cell viability following treatment with 0, 1, 2.5, 5, 10 or 100 μM CoQ10. Data are presented as the mean ± SEM from 6 independent experiments. Statistical significance was assessed using one‐way ANOVA followed by Dunnett's multiple‐comparisons test against the untreated control. **p < 0.01 and ****p < 0.0001 versus the untreated control.
**Figure S4:** Animal allocation and assay‐specific sample sizes. A total of 84 male C57BL/6J mice were stratified into six pretreatment age cohorts of 1, 9, 15, 19, 21 and 23 months, with 14 mice per cohort. Within each age cohort, mice were allocated to the control and CoQ10‐treated groups, with seven mice per treatment group. Following the 12‐week intervention, peripheral blood and bone marrow samples were collected at 4, 12, 18, 22, 24 and 26 months of age. Circulating biomarker and platelet‐function analyses included seven mice per age‐by‐treatment group. Plasma CoQ10 measurements, megakaryocyte proliferation, maturation and polyploidy analyses, and Western blotting included six mice per age‐by‐treatment group. Quantitative proteomic analysis included six mice from each 24‐month‐old treatment group. Each mouse constituted one independent biological replicate, and samples were not pooled.

**Table S1:** Proteomics results and accompanying data annotations.

## Data Availability

The original contributions presented in this study are included in the article/Supporting Information. Further inquiries can be directed to the corresponding authors.
