# Supplementary material for: Dietary CoQ10 Ameliorates Age‐Related Megakaryocyte Dysfunction via COPS3‐Mediated Activation of Autophagy
Source: Aging Cell. 2026 Sep 29;25(10):e70726. doi: 10.1111/acel.70726 (PMC13621815; doi:10.1111/acel.70726)
Supplement: Supplementary file 1 [file ACEL-25-e70726-s002.docx]

**Figure S1.** Coenzyme Q10 reduces oxidative stress levels in naturally aging mice.

Plasma(A): MDA; (B):SOD; (C):CAT; (D):GSF level(n=7). Data are presented as mean ± SEM. a: *P*<0.05 compared with 4-month-old group (4M);b: *P*<0.05 compared with 12-month-old group (12M);c: *P*<0.05 compared with 18-month-old group (18M);*:*P*<0.05 Compared with the same age group.

**Figure S2.** Coenzyme Q10 supplementation increases ubiquinol and ubiquinone reduced CoQ10 levels in naturally aged mice

Plasma(A): ubiquinol; (B): ubiquinone level (n=6). Data are presented as mean ± SEM. a: *P*<0.05 compared with 4-month-old group (4M)*:*P*<0.05 Compared with the same age group.

**Figure S3**：**Dose-response validation for the selection of H₂O₂ and CoQ10 concentrations in MEG-01 cells.**

(A–D) Representative bright-field images of SA-β-gal staining in MEG-01 cells treated with 0, 1, 10 or 100μM H₂O₂ for 7day. Insets show magnified views of the boxed regions. Scale bars, 50 μm. (E) Quantification of SA-β-gal-positive cells following H₂O₂ treatment. (F) Cell viability following treatment with 0, 1, 10, 100, 200 or 500 μM H₂O₂. (G) Cell viability following treatment with 0, 1, 2.5, 5, 10 or 100 μM CoQ10. Data are presented as the mean ± SEM from 6 independent experiments. Statistical significance was assessed using one-way ANOVA followed by Dunnett’s multiple-comparisons test against the untreated control. ***P*<0.01 and *****P*<0.0001 versus the untreated control.

**Figure S4：Animal allocation and assay-specific sample sizes.**

A total of 84 male C57BL/6J mice were stratified into six pretreatment age cohorts of 1, 9, 15, 19, 21 and 23 months, with 14 mice per cohort. Within each age cohort, mice were allocated to the control and CoQ10-treated groups, with seven mice per treatment group. Following the 12-week intervention, peripheral blood and bone marrow samples were collected at 4, 12, 18, 22, 24 and 26 months of age. Circulating biomarker and platelet-function analyses included seven mice per age-by-treatment group. Plasma CoQ10 measurements, megakaryocyte proliferation, maturation and polyploidy analyses, and Western blotting included six mice per age-by-treatment group. Quantitative proteomic analysis included six mice from each 24-month-old treatment group. Each mouse constituted one independent biological replicate, and samples were not pooled.
